# Forecast for the zone of viticulture in European Russia under climate change

**DOI:** 10.18699/VJGB-22-33

**Published:** 2022-05

**Authors:** L.Yu. Novikova, P.V. Ozerski

**Affiliations:** Federal Research Center the N.I. Vavilov All-Russian Institute of Plant Genetic Resources (VIR), St. Petersburg, Russia; Federal Research Center the N.I. Vavilov All-Russian Institute of Plant Genetic Resources (VIR), St. Petersburg, Russia

**Keywords:** viticulture, climatic limiting factors, climate change, trends, forecast, GIS, виноградарство, лимитирующие климатические факторы, изменения климата, тренды, прогнозы, ГИС

## Abstract

Climate warming has turned out to be a significant factor in viticulture and winemaking in all grape-growing areas of the world. Many countries consider the advance of viticulture to the north and to mountainous areas as a possible way to adapt to warming. The factors limiting the zone of viticulture in Russia have been identified by Soviet scientist F.F. Davitaya in 1948, and they are still relevant. They are the sum of active temperatures above 10 °C (ΣT10 > 2500 °C), mean of absolute minimum temperatures (Tmin > –35 °C), length of the frost-free period (Lff < 150 days), and hydrothermal coefficient (0.5 < HTC <2.5). The values of these limiting factors in the present-day zone of commercial viticulture (ZCV) correspond to the ranges defined by F.F. Davitaya, with the exception of Tmin, which in the modern ZCV in European Russia is above –26 °C everywhere. The objective of this work was to assess the possibility of moving the boundaries of the ZCV to the north under the existing and predicted climate conditions in European Russia. The 1980–2019 daily data from 150 weather stations of the Federal Service for Hydrometeorology and Environmental Monitoring were used to calculate mean long-term values, trends and forecasts for 2050 for the ZCV limiting factors and locate the points lying in the range acceptable for viticulture. The QGIS program was applied to plot the points on the European Russia map and mark the terminal latitude. Versions with Tmin > –26 °C and Tmin > –35 °C were considered. On average for European Russia, in 1980–2019, there was an increase in ΣT10 , Tmin, and Lff and a decrease in HTC. However, in the same period, Tmin showed a tendency toward decreasing at a number of points at latitudes lower than 55° N. The increase in heat supply during the growing season in European Russia implies a possibility of expanding the ZCV northward, beyond the present-day terminal latitude of 46.6° N, to 51.8° N under the existing conditions, and up to 60.7° N by 2050. In addition, even under the current conditions viticulture is possible in the area of Kaliningrad (54° N, 20° E). Using extra protective measures in winters not colder than –35 °C would make it possible to grow grapes at up to 53.3° N under the current conditions and at up to 60.7° N under the prognosticated ones. At the same time, a possible decrease in the minimum winter temperature at the south of European Russia will require additional protective measures in winter, while an increase in the aridity of the climate on the northwest coast of the Caspian Sea will reduce the area under non-irrigated vineyards.

## Abstract (ru)

Потепление климата оказалось существенным фактором для виноградарства и виноделия всех виноградарских районов мира. Многие страны рассматривают продвижение виноградарства на север и в горные районы как возможный путь адаптации к потеплению. Факторы, лимитирующие зону виноградарства в России, определены советским ученым Ф.Ф. Давитая в 1948 г. и актуальны до сих пор. Это сумма активных температур выше 10 °С (ΣT10 > 2500 °C), средний из абсолютных минимумов температуры (Tmin > –35 °C), продолжительность безморозного периода (Lff < 150 сут) и гидротермический коэффициент (0.5 < ГТК < 2.5). Значения лимитирующих факторов современной зоны промышленного виноградарства (ЗПВ) соответствуют определенным Ф.Ф. Давитая диапазонам, за исключением Тmin, которая в современной ЗПВ на европейской территории России везде выше –26 °C. Целью исследования было определение возможности продвижения на север границ зоны промышленного виноградарства в современных и прогнозируемых климатических условиях европейской территории России. По суточным данным 1980–2019 гг. для 150 метеостанций Росгидромета рассчитали среднемноголетние значения, тренды и прогнозы к 2050 г. значений лимитирующих факторов ЗПВ, определили точки, лежащие в допустимом для виноградарства диапазоне. В программе QGIS нанесли точки на карту европейской территории России, определили предельную широту. Были рассмотрены варианты с Тmin > –26 °C и Tmin > –35 °C. В 1980–2019 гг. в среднем на европейской территории России наблюдался рост ΣT10, Tmin, Lff и снижение ГТК. Однако южнее 55° N в ряде точек прослеживалась тенденция к снижению Tmin . Рост теплообеспеченности вегетационного периода на европейской территории России создает предпосылки продвижения промышленного виноградарства к северу от современной предельной широты 46.6° до 51.8° в текущих условиях, а к 2050 г. – до 60.7° N. Кроме того, уже сейчас виноградарство возможно в районе Калининграда (54° N, 20° E). При дополнительных мерах по укрытию на зиму до –35 °С виноградарство возможно до 53.3° N в текущих условиях и до 60.7° N – в прогнозируемых. Возможное снижение минимальной температуры зимы на юге европейской территории России потребует дополнительных мер защиты зимой, а повышение засушливости климата на северо-западном побережье Каспийского моря будет уменьшать площади под неорошаемыми виноградниками.

## Introduction

Climate zones suited for high-quality viticulture and winemaking are narrow and greatly depend on the effect of climate change (Hannah et al., 2013; Mozell, Thach, 2014; Santos et al., 2020). A more than 1 °C temperature rise during the small climatic optimum in the 8th–13th centuries led to the advance of the viticulture border in Western and Central Europe to the north by 3–4° N, but from the 15th century the grape-growing area shifted southward (Barash, 1989; Khromov, Petrosyants, 2012). At present, the area under viticulture in the Northern hemisphere is confined between 30° and 50° N, corresponding to the limits of the mean April-to-October temperature of 12–22 °C, or 13–21 °C for high-quality wine production (Schultz, Jones, 2010; Jones, 2012).

According to forecasts, future warming, on the one hand, will produce a beneficial effect on viticulture as a result of the inclusion of new areas, but, on the other hand, will generate serious problems in the areas of traditional viticulture (Roy et al., 2017; Hewer, Brunette, 2020; Vyshkvarkova, Rybalko, 2021). By 2050, the area suitable for viticulture in main winemaking regions is expected to decrease by 19–62 %, as predicted by the RCP 4.5 global climate change scenario, or by 25–73 % according to the RCP 8.5 scenario (Hannah et al., 2013). Contemporary climate change initiates the shift of the zone of commercial viticulture to the north and to mountainous regions (Jones, 2012; Vršič, Vodovnik, 2012; Hannah et al., 2013; Mozell, Thach, 2014; Quénol et al., 2014). Russia is among the countries that may face expressly significant consequences of climate warming (Houtan et al., 2021).

To predict the impact of climate change on the efficiency of viticulture in grape-growing regions, their climate resources are assessed using various indicators, such as the sums of active and effective temperatures, biologically active sums of effective temperatures, Winkler index, mean April-to-October temperatures, spring frost risk index, aridity index, cold index, Huglin and Branas heliothermal indices, Selyaninov’s hydrothermal coefficient, etc. (Lorenzo et al., 2013; Blanco-Ward et al., 2019; Rybalko, 2020; Pipan et al., 2021; Vyshkvarkova, Rybalko, 2021). The limiting factors in viticulture are not the same under different environmental and geographic conditions. The most important requirements for grapevine cultivation are temperature and light during the active growing season. In arid regions, rainfall becomes a limiting factor, so irrigation is introduced. Close to the northern boundaries of commercial viticulture, it is limited by winter conditions (Likhovskoi et al., 2016; Roy et al., 2017).

F.F. Davitaya (Davitaya, 1948) analyzed the world’s viticulture zones in the first third of the 20th century and made a comprehensive assessment of the range of climatic requirements for grapes, highlighting the characteristics that were relevant for the USSR: the temperature in the beginning and end of the growing season was 10 °C; the sum of temperatures above 10 °C during the growing season (ΣT10) was higher than 2500 °C; the inhibitory high temperature was 35–40 °C; the required minimum mean temperature of the warmest month was 16–18 °С, or 17–19 °С for high-quality wine production; the length of the frostfree period (Lff) was at least 150 days; the mean of absolute minimum temperatures (Tmin) for uncovered grapevine cultivation was not lower than –15 °C or, with conventional ways of protection from the cold, –35 °C; and Selyaninov’s hydrothermal coefficient (HTC) was within the range from 0.5 to 1.5–2.5 (Davitaya, 1948, p. 172–174). This system of indicators is still valid (Mishchenko, 2009; Roy et al., 2017; Hewer, Brunette, 2020).

Geographic areas with a climate that is currently suitable for growing certain crops or may become so in the future are visualized using GIS techniques (Hannah et al., 2013; Nesbitt et al., 2018). Such approach also makes it possible to identify and adjust the parameters of the climatic niche for a species, i. e., the range of agroclimatic parameters under which its development is possible. For this purpose, data from definite geographic points where this species occurs are analyzed (Soberon, Nakamura, 2009; Peterson et al., 2015; Wójtowicz M., Wójtowicz A., 2020).

**Fig. 1.**
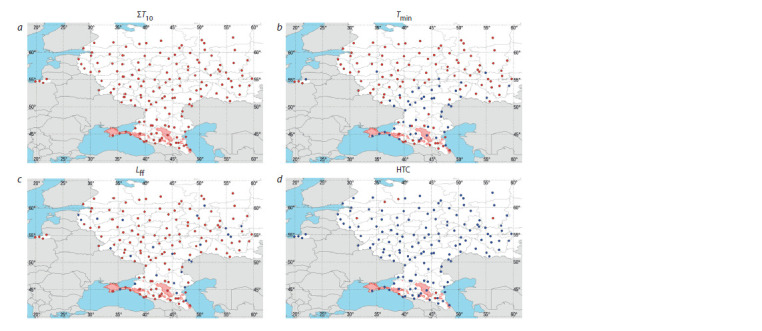
Trends of the limiting factors for viticulture recorded at 150 weather stations in European Russia: a, the sum of temperatures above 10 °С; b, the absolute minimum of temperature; c, the length of the frost-free period; d, HTC. Red dots are positive trends, and blue dots are zero or negative trends. The zone of commercial viticulture in the early 21st century is shaded. The ZCV map was taken from the AgroAtlas resource (AgroAtlas, 2008) and modified.

In Russia, the zone of commercial viticulture (ZCV) is located between the Black, Azov and Caspian seas and in the Crimea at the latitudes of 41.6–46.6° N and the longitudes of 32.5–48.5° E (AgroAtlas, 2008) (Fig. 1). The range of climate characteristics for the ZCV in European Russia (ER) in the early 21st century has mostly remained within the limits outlined by F.F. Davitaya (1948), significantly deviating from them in only one indicator – the minimum winter temperature, which in the present-day ZCV zone does not fall below –26 °C (Chistyakov, Novikova, 2020). The main limitation for the advance of grapevine cultivation to the north is Tmin, and it is low HTC that limits its shift to the northwestern coast of the Caspian Sea. If we accept the possibility of cultivation at Tmin > –35 °C, then ΣT10 and Lff would become limiting factors in the north

Our subject of interest is the advance of grapevine to the north, so the limitations imposed by temperatures above 35–40 °C are not discussed (Leewen et al., 2013). July temperatures above 16 °C are observed in ER to the south of 60–63° N; this factor is also not limiting and is not considered below. Thus, ΣT10, Lff , Tmin, and HTC are the limiting factors for the ZCV in ER when viticulture is moving northward. A deficit in moisture supply (the requirement is HTC > 0.5) limits non-irrigated viticulture on the northeastern coast of the Caspian Sea.

The objective of this work was to assess the possibility of moving the boundaries of the ZCV to the north under the existing and predicted climate conditions in ER.

## Materials and methods

Conventionally, the ER territory is regarded here as limited by 63° N and 60° E. The software product applied was QGIS 3.22.01. The analysis of the climate in ER was performed pointwise, according to the data of 150 weather stations of the Federal Service for Hydrometeorology and Environmental Monitoring, with more than 20 years of observations in the period of 1980–2019. We used daily data from an open web source (RIHMI – World Data Center, 2020)2. The VITIS TIME SERIES program (Novikova, Lebedeva, 2019) was used to calculate the values of ΣT10, Tmin, Lff and HTC for each point for each year and their trends for the period of 1980–2019.

Values average for 1980–2019 were attributed to 2000, individual ΣT10, Lff , Tmin and HTC forecasts for 2050 were calculated for each point, and points where grapevine cultivation is possible were identified according to the set of requirements proposed by F.F. Davitaya (1948) for protected viticulture and taking into account specific features of the modern Russian viticulture with Tmin < –26 °C. The study adopted a significance level of 5 %.

## Results

Changes of climatic factors important for viticulture in ER during 1980–2019

In 1980–2019, on average, ER showed an increase in ΣT10, Tmin and Lff , and a decrease in HTC. The average trend for 150 stations was: ΔΣT10 = 11.52 °С/year, ΔTmin= 0.02 °С/year, ΔLff = 0.31 days/year, and ΔHTC = –0.01 units/year (see the Table).

**Table 1.**
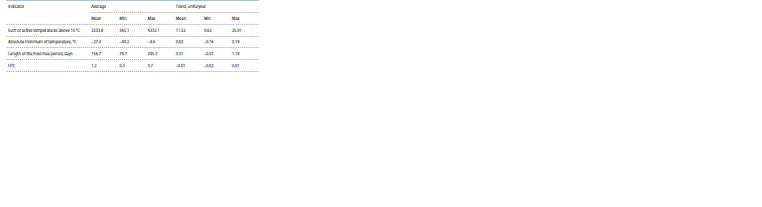
Trends of the limiting factors in the zone of commercial viticulture at 150 weather stations in ER in 1980–2019

With the average tendency for the studied stations to increase Tmin, the trend was zero or negative at 48 points.

There were only 8 statistically significant Tmin trends, and two of them were negative. The Tmin trend increased with the latitude (r = 0.52), i. e., warming in winter was more intense in the north of ER; out of 61 points located to the north of 55° N, a negative trend was observed only at three points (see Fig. 1, b).

Lff increased on average; however, negative trends were observed at 25 points out of 150 studied. There were only 40 significant trends, and positive values were registered in 39 of them.

With the average tendency toward a decrease in HTC, induced by an active increase in temperatures and, on average, the absence of a tendency toward changes in rainfall, the HTC values increased at 8 points. The HTC trends were significant at 20 points, all of them being negative.

Potential zone of commercial viticulture

**Fig. 2.**
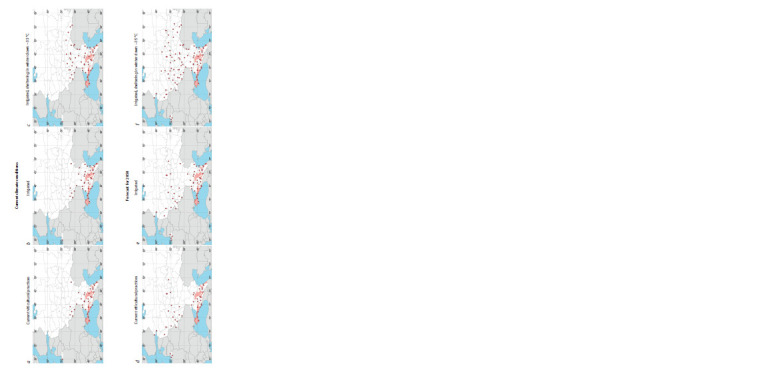
Points in European Russia where climate conditions are suitable for grapevine cultivation with existing viticultural practices (a, d ), irrigation (b, e), sheltering in winters down to –35 °С (c, f ) under the present-day conditions and by 2050 The zone of commercial viticulture in the early 21st century is shaded.

Under the present-day climate conditions, defined as the average values of the limiting factors for the period of 1980–2019, grapevine cultivation without irrigation and with usual protective measures for the winter season is possible at 36 points out of 150 studied (Fig. 2, a), including one point in Kaliningrad Province (Baltiysk, in 1980–2019: ΣT10 = 2567 °C, Lff = 229 days, Tmin = –15 °C, HTC = 1.2). If we add the points where insufficient moisture (HTC < 0.5) can be compensated by irrigation, then their number will reach 41 (see Fig. 2, b). If we add areas with winter temperature minima reaching –35 °С, then the number of points will increase to 58 (see Fig. 2, c). The heat supply during the growing season in ER implies a possibility to move grapevine cultivation to the north of the current terminal latitude of the ZCV (46.6° N) up to 51.8° N even now. With additional sheltering measures for the winter season with temperatures down to –35 °C, it can be moved to 53.3° N.

By 2050, wintering conditions are expected to worsen in the south of ER, i. e., in a number of places the minimum winter temperature will drop below –26 °C. However, due to the movement of heat to the north, the number of points suitable for commercial grapevine cultivation will increase to 43. Additional viticultural practice measures will increase this number even more – up to 56, with the inclusion of irrigated vineyards (see Fig. 2, e), and up to 95, with the inclusion of points with temperatures in winter above –35 °C (see Fig. 2, f). The increase of heat supply during the growing season creates the prerequisites for the advance of viticulture to 60.7° N by 2050. St. Petersburg may enter the zone of viticulture: by 2050, the forecasts for the area are: ΣT10 = 2772 °C, Tmin = –18 °C, Lff = 191 days, and HTC = 1.4. Without the Baltic Sea coast, the northernmost point among the forecasted ones is located on the latitude of 58.1° N.

## Discussion

The pace of climate change and anomalies in the harvests of the world’s staple crops are on the rise (Jägermeyr et al., 2021). However, the changes in agroclimatic indicators have regional specificities (Sirotenko et al., 2013; Hewer, Brunette, 2020), which was confirmed in the process of this study. Since the 1970s, the sums of active temperatures have been increasing in all ER regions, and more actively in the south; however, the January temperature increases faster in northern latitudes. Total rainfalls have both positive and negative tendencies (Sirotenko et al., 2013). Changes accelerated in the early 21st century: for example, from 1975 to 2004 the HTC reduced in most of ER, with the exception of several regions, and in 1980–2019 we observed that the forecasts concerning the growth of aridity throughout ER came true (Sirotenko, Pavlova, 2009). Warming is accompanied by intensification of climate instability. In 2007, a decrease in the number of winters with threatening drops in air temperature was predicted (The Economics of Climate Change, 2006), but according to the data of 1980–2019 the minimum temperature and the number of dangerous frosts in winter did not decrease despite the rise of the mean winter temperature. The tendency toward an increase in the frequency of extreme events and a decrease in the absolute minimum of annual temperature was observed in many regions of the world under global warming (Bucur et al., 2019).

The study of climate change near the northern border of ER’s zone of commercial viticulture – at the Don Ampelographic Collection named after Y.P. Potapenko – also showed that in 1981–2017 the sum of temperatures above 10 °С increased (by 170 °С/10 years), the total rainfall during the period of active growth decreased (by 21 mm/10 years), the mean temperature of the winter dormancy period grew (by 0.5 °С/10 years), the length of the frost-free period extended (by 0.7 days/year), while the number of winter days with temperatures below –20 °C and the minimum winter temperature did not change (Novikova, Naumova, 2018, 2019).

Assessments of the climatic requirements of grapes, made by F.F. Davitaya in the 1930s on the basis of the world’s experience in viticulture, turned out to be relevant for ER in the early 21st century. Despite the fact that there are grapevine varieties with temperature requirements of 2100 °С and lower (Mishchenko, 2009; Naumova, Novikova, 2015), the ZCV in ER is limited to temperature sums of 2500 °С, which is explained by the need to have 80–90 % of years with the sums of temperatures necessary for profitable commercial cultivation of a variety (Losev, Zhurina, 2004). There is also concordance between the ranges of other climatic factors, with the exception of the minimum winter temperature: in the contemporary ZCV in ER it is –26 °C versus –35 °С reported by F.F. Davitaya. Values of the limiting factors for the zone of viticulture in the Canadian province of Quebec are also close to F.F. Davitaya’s estimates: Lff > 150 days, the sum of effective April-to-October temperatures above 10 °С are DD10 > 900 °С, Tmin > –34 °C, and the annual number of very cold days (T < –22 °C) is less than 30 (Roy et al., 2017). Canadian researchers (Hewer, Brunette, 2020) rank the territories according to the minimum winter temperatures reflecting the degree of suitability for viticulture: –34…–30 °C means poorly suitable conditions; –30…–27 °C medium; –27…–22 °C good, and >–22 °C very good. Thus, the considered options with temperature limits Tmin > –35 °С and Tmin > –26 °С correspond to different degrees of risk and economic efficiency of viticulture. The boundary of Tmin > –35 °C, reported by F.F. Davitaya, possibly corresponds to amateur viticulture.

The ongoing climate change affects all grapevine traits (Vršič, Vodovnik, 2012; Novikova, Naumova, 2019, 2020) and requires adaptation of viticulture and winemaking in all viticultural regions of the world (White et al., 2006; Schultz, Jones, 2010; Jones, 2012; Hannah et al., 2013; Quénol et al., 2014; Bardaji, Iraizoz, 2015). Many countries regard moving northward and into mountainous areas as a possible way for viticulture to adapt to warming (White et al., 2006; Hannah et al., 2013; Schultze et al., 2016; Tóth, Végrári, 2016; Roy et al., 2017; Vyshkvarkova, Rybalko, 2021). Our calculations have shown that a significant advance of viticulture in ER to the north from the current latitude of 46.6° N is possible: even under the existing climate conditions grapevine cultivation could be extended to Kaliningrad and by 2050 to Leningrad Province. During the maximum warming in the 12–13th centuries, well-developed viticulture was underway on the Baltic coast as well as in England (Khromov, Petrosyants, 2012).

However, the trends of 1980–2019 show a decrease in the minimum winter temperature in the southern regions of ER, which may make viticulture less profitable there due to the need for additional winter sheltering measures.

For the main viticulture regions of the world, a decrease in rainfall and an increase in high temperatures (Biasi et al., 2019; Santos et al., 2020) become risk factors and enhance the need for irrigation (Hall et al., 2016; Chrysargyris et al., 2020). The HTC decrease throughout ER is limiting non-irrigated viticulture to the north of the Caspian Sea, where climate aridity will grow. For the rest of ER, moisture conditions remain favorable. A study conducted by Crimean colleagues confirms that viticulture in the vicinity of Sevastopol in the 21st century will be possible without irrigation, but grapevines may experience moisture deficiency (Vyshkvarkova et al., 2021).

Short-term adaptation measures should focus on specific threats, mainly changes in crop management practices (e. g., irrigation, sunscreens to protect leaves, etc.). Further, the change in the composition and taste of grapes and wine will cause regional changes in the assortment of varieties and style of winemaking (Mira de Orduña, 2010; Fraga, Santos, 2017) and stimulate the advance of viticulture to the northern and mountainous regions.

We did not consider the negative effect of rising high temperatures, since the southern boundary of the ZCV in Russia lies at the latitude of 41.6° N, higher than the southern boundary of the world’s viticulture (30° N). However, the threat of excessively high temperatures in the south of the ZCV will remain in the future. In addition, the most important issue of matching the quality of soils in ER to the needs of viticulture has not been considered. These aspects require further research.

## Conclusion

The increase of heat supply during the growing season in ER implies a possibility to expand the zone of commercial viticulture northward from the current terminal latitude (46.6° N) up to 51.8° N and by 2050 to 60.7° N. Besides, even under the existing conditions it is possible to develop viticulture in the area of Kaliningrad (54° N, 20° E). Additional winter sheltering measures at temperatures down to –35 °С would make it possible to cultivate grapevine up to 53.3° N under the current conditions and up to 60.7° N under the predicted conditions. The increasing aridity of the climate on the northwestern coast of the Caspian Sea will reduce the area under non-irrigated vineyards. A possible drop of the minimum winter temperature in the south of ER will require additional protective measures during the winter season.

## Conflict of interest

The authors declare no conflict of interest.

## References

AgroAtlas. Interactive Agricultural Ecological Atlas of Russia and
Neighboring Countries. Economic Plants and their Diseases, Pests
and Weeds. 2008. [Electronic resource]. http://www.agroatlas.ru/en/
index.html (Accessed August 20, 2021).

Barash S.I. History of Bad Harvests and Weather in Europe. Leningrad:
Hydrometeoizdat Publ., 1989. (in Russian)

Bardaji I., Iraizoz B. Uneven responses to climate and market influencing
the geography of high-quality wine production in Europe. Reg.
Environ. Change. 2015;15:79-92. DOI 10.1007/s10113-014-0623-y.

Biasi R., Brunori E., Ferrara C., Salvati L. Assessing impacts of climate
change on phenology and quality traits of Vitis vinifera L.: the
contribution of local knowledge. Plants. 2019;8:121. DOI 10.3390/
plants8050121.

Blanco-Ward D., Ribeiro A.C., Barreales D., Castro J., Verdial J.,
Feliciano M., Viceto C., Rocha A., Carlos C., Silveira C., Miranda
A. Climate change potential effects on grapevine bioclimatic
indices: a case study for the Portuguese demarcated Douro Region
(Portugal). BIO Web of Conf. 2019;12:01013. DOI 10.1051/
bioconf/20191201013.

Bucur G.M., Cojocaru G., Antoce A.O. The climate change influences
and trends on the grapevine growing in Southern Romania: a longterm study, 42nd World Congress of Vine and Wine. BIO Web Conf.
2019;15:01008. DOI 10.1051/bioconf/20191501008

Chistyakov P.N., Novikova L.Yu. Evaluation of the possibility of moving
northward of the zone of grape cultivation in the ETR. In: Book
of abstracts of the All-Russian sci. conf. with international participation
“Contribution of Agrophysics to Solving Fundamental Problems
of Agricultural Science”, St. Petersburg, October 1–2, 2020.
St. Petersburg: FGBNU AFI, 2020;275-281. (in Russian)

Chrysargyris A., Xylia P., Litskas V., Stavrinides M., Heyman L., Demeestere
K., Höfte M., Tzortzakis N. Assessing the impact of drought
stress and soil cultivation in Chardonnay and Xynisteri grape cultivars.
Agronomy. 2020;10:670. DOI 10.3390/agronomy10050670.

Davitaya F.F. Climatic Zones of Grapes in the USSR. Moscow:
Pishchepromizdat Publ., 1948. (in Russian)

Fraga H., Santos J.A. Daily prediction of seasonal grapevine production
in the Douro wine region based on favourable meteorological conditions.
Aust. J. Grape Wine Res. 2017;23:296-304. DOI 10.1111/
ajgw.12278.

Hall A., Mathews A.J., Holzapfel B. Potential effect of atmospheric
warming on grapevine phenology and post-harvest heat accumulation
across a range of climates. Int. J. Biometeorol. 2016;60(9):1405-
1422. DOI 10.1007/s00484-016-1133-z.

Hannah L., Roehrdanz P.R., Ikegami M., Shepard A.V., Shaw M.R.,
Tabor G., Zhi L., Marquet P.A., Hijmans R.J. Climate change, wine,
and conservation. Proc. Natl. Acad. Sci. USA. 2013;110(17):6907-
6912. DOI 10.1073/pnas.1210127110.

Hewer M., Brunette M. Climate change impact assessment on grape
and wine for Ontario, Canada’s appellations of origin. Reg. Environ.
Change. 2020;20(3):86. DOI 10.1007/s10113-020-01673-y.

Houtan K.S., Tanaka K.R., Gagné T.O., Becker S.L. The geographic
disparity of historical greenhouse emissions and projected climate
change. Sci. Adv. 2021;7:eabe4342. DOI 10.1126/sciadv.abe4342

Jägermeyr J., Müller C., Ruane A.C., Elliott J., Balkovic J., Castillo
O., Faye B., Foster I., Folberth C., Franke J.A., Fuchs K., Guarin
J.R., Heinke J., Hoogenboom G., Iizumi T., Jain A.K., Kelly D.,
Khabarov N., Lange S., Lin T.-S., Liu W., Mialyk O., Minoli S.,
Moyer E.J., Okada M., Phillips M., Porter C., Rabin S.S., Scheer C.,
Schneider J.M., Schyns J.F., Skalsky R., Smerald A., Stella T., Stephens
H., Webber H., Zabel F., Rosenzweig C. Climate impacts on
global agriculture emerge earlier in new generation of climate and
crop models. Nat. Food. 2021;2:873-885. DOI 10.1038/s43016-
021-00400-y.

Jones G. Climate, grapes, and wine: structure and suitability in a
changing climate. Acta Hort. 2012;931:19-28. DOI 10.17660/
ActaHortic.2012.931.1.

Khromov S.P., Petrosyants M.A. Meteorology and Climatology. Moscow:
Moscow State University Publ., 2012. (in Russian)

Leewen C., Schultz H., de Cortazar-Atauri I.G., Duchêne E., Ollat N.,
Pieri P., Bois B., Goutouly J.-P., Quénol H., Touzard J.-M., Malheiro
A.C., Bavaresco L., Delrot S. Why climate change will not
dramatically decrease viticultural suitability in main wine-producing
areas by 2050. Proc. Natl. Acad. Sci. USA. 2013;110(33):3051-3052.
DOI 10.1073/pnas.1307927110.

Likhovskoi V.V., Zlenko V.A., Volinkin V.A., Oleinikov N.P., Polylyax
A.A., Vasylyk I.A., Troshin L.P. Frost resistance of Crimean
indigenous grape varieties and their hybrids. Nauchnyy Zhurnal
KubGAU = Scientific Journal of KubSAU. 2016;117(03):681-694.
(in Russian)

Lorenzo M.N., Taboada J.J., Lorenzo J.F., Ramos A.M. Influence of
climate on grape production and wine quality in the Rías Baixas,
north-western Spain. Reg. Environ. Change. 2013;13:887-896.
DOI 10.1007/s10113-012-0387-1.

Losev A.P., Zhurina L.L. Agrometeorology. Moscow: KolosS Publ.,
2004. (in Russian)

Mira de Orduña R. Climate change associated effects on grape and
wine quality and production. Food Res. Int. 2010;43:1844-1855.
DOI 10.1016/j.foodres.2010.05.001.

Mishchenko Z.A. Agro-climatology. Kiev: KNT Publ., 2009. (in Russian)

Mozell M.R., Thach L. The impact of climate change on the global wine
industry: challenges & solutions. Wine Econ. Policy. 2014;3(2):81-
89. DOI 10.1016/j.wep.2014.08.001.

Naumova L.G., Novikova L.Yu. Temperature analysis of interphase
periods of grape varieties of the collection of the All-Russian Scientific
Research Institute of Viticulture and Winemaking named after
Ya.I. Potapenko. Vinodelie i Vinogradarstvo = Wine-making and Viticulture.
2015;5:46-50. (in Russian)

Nesbitt A., Dorling S., Lovett A. A suitability model for viticulture in
England and Wales: opportunities for investment, sector growth and
increased climate resilience. J. Land Use Sci. 2018;13(4):414-438.
DOI 10.1080/1747423X.2018.1537312.

Novikova L.Yu., Lebedeva E.G. Certificate of state registration of the
computer program ‘Program for predicting the reaction of grape varieties
to climate change VITIS TIME SERIES’ No. 2019664805
dated November 13, 2019. (in Russian)

Novikova L.Yu., Naumova L.G. Regression analysis of winter hardiness
of grape cultivars from Ya.I. Potapenko Don ampelographic
collection. Magarach. Vinogradarstvo i Vinodelie = Magarach. Viticulture
and Winemaking. 2018;4:59-61. (in Russian)

Novikova L.Yu., Naumova L.G. Structuring ampelographic collections
by phenotypic characteristics and comparing the reaction of grape
varieties to climate change. Vavilovskii Zhurnal Genetiki i Selektsii =
Vavilov Journal of Genetics and Breeding. 2019;23(6):772-779.
DOI 10.18699/VJ19.551.

Novikova L.Y., Naumona L.G. Dependence of fresh grapes and wine
taste scores on the origin of varieties and weather conditions of the
harvest year in the northern zone of industrial viticulture in Russia.
Agronomy. 2020;10(10):1613. DOI 10.3390/agronomy10101613.

Peterson A.T., Papeş М., Soberón J. Mechanistic and correlative
models of ecological niches. Eur. J. Ecol. 2015;1(2):28-38. DOI
10.1515/eje-2015-0014.

Pipan P., Hall A., Rogiers S.Y., Holzapfel B.P. Accuracy of interpolated
versus in-vineyard sensor climate data for heat accumulation modelling
of phenology. Front. Plant Sci. 2021;12:635299. DOI 10.3389/
fpls.2021.635299.

Quénol H., Grosset M., Barbeau G., van Leeuwen C., Hofmann M.,
Foss C., Irimia L., Rochard J., Boulanger J.-P., Tissot C., Miranda C.
Adaptation of viticulture to climate change: high resolution observation
of adaptation scenario for viticulture: the ADVICLIM European
project. Bull. de l’OIV. 2014;87(1001-1002-1003):395-406.

Roy P., Grenier P., Barriault E., Logan T., Blondlot A., Bourgeois G.,
Chaumont D. Probabilistic climate change scenarios for viticultural
potential in Québec. Clim. Change. 2017;143(1):43-58.
DOI 10.1007/s10584-017-1960-x.

Rybalko E.A. Climatic indices in viticulture. Magarach. Vinogradarstvo
i Vinodelie = Magarach. Viticulture and Winemaking.
2020;22(1):26-28. DOI 10.35547/iM.2020.22.1.005. (in Russian)

Santos J.A., Fraga H., Malheiro A.C., Moutinho-Pereira J., Dinis L.-T.,
Correia C., Moriondo M., Leolini L., Dibari C., Costafreda-Aumedes
S., Kartschall T., Menz C., Molitor D., Junk J., Beyer M., Schultz
H.R. A review of the potential climate change impacts and adaptation
options for European viticulture. Appl. Sci. 2020;10(9):3092.
DOI 10.3390/app10093092.

Schultz H.R., Jones G.V. Climate induced historic and future changes
in viticulture. J. Wine Res. 2010;21:137-145. DOI 10.1080/
09571264.2010.530098.

Schultze S.R., Sabbatini P., Luo L. Effects of a warming trend on cool
climate viticulture in Michigan, USA. SpringerPlus. 2016;5(1):1119.
DOI 10.1186/s40064-016-2777-1.

Sirotenko O.D., Abashina E.V., Pavlova V.N. Dynamics of climate-conditioned
changes in heat supply, moisture content and productivity
of the agricultural zone of Russia. Trudy FGBU VNIISHM = Proceedings
of the FSBI VNIISHM. 2013;38:41-53. (in Russian)

Sirotenko O.D., Pavlova V.N. The impact of climate change on agriculture.
In: Development of Agricultural Meteorology in Russia.
Obninsk, 2009;168-175. (in Russian)

Soberon J., Nakamura M. Niches and distributional areas: concepts,
methods and assumptions. Proc. Natl. Acad. Sci. USA.
2009;106:19644-19650. DOI 10.1073/pnas.0901637106.

The Economics of Climate Change. The Stern Review. Nicholas Stern.
Cabinet Office – HM Treasury, UK, 2006.

Tóth J.P., Végvári Z. Future of wine grape growing regions in Europe.
Aust. J. Grape Wine Res. 2016;22:64-72. DOI 10.1111/ajgw.12168.

Vršič S., Vodovnik T. Reactions of grape varieties to climate changes
in North East Slovenia. Plant Soil Environ. 2012;58(1):34-41.
DOI 10.17221/352/2011-PSE.

Vyshkvarkova E., Rybalko E. Forecast of changes in air temperatures
and heat indices in the Sevastopol region in the 21st century and
their impacts on viticulture. Agronomy. 2021;11:954. DOI 10.3390/
agronomy11050954.

Vyshkvarkova E., Rybalko E., Marchukova O., Baranova N. Assessment
of the current and projected conditions of water availability in the
Sevastopol region for grape growing. Agronomy. 2021;11(8):1665.
DOI 10.3390/agronomy11081665.

White M.A., Diffenbaugh N.S., Jones G.V., Pal J.S., Giorgi F. Extreme
heat reduces and shifts United States premium wine production
in the 21st century. Proc. Natl. Acad. Sci. USA. 2006;103:11217-
11222. DOI 10.1073/pnas.0603230103.

Wójtowicz M., Wójtowicz A. the effect of climate change on linolenic
fatty acid in oilseed rape. Agronomy. 2020;10(12):2003. DOI
10.3390/agronomy10122003.

